# Mineral Concentrations in Bovine Milk from Farms with Contrasting Grazing Management

**DOI:** 10.3390/foods10112733

**Published:** 2021-11-09

**Authors:** Sokratis Stergiadis, Nanbing Qin, Gergely Faludi, Stephane Beauclercq, Joe Pitt, Natasa Desnica, Ásta H. Pétursdóttir, Eric E. Newton, Angelos E. Angelidis, Ian Givens, David J. Humphries, Helga Gunnlaugsdóttir, Darren T. Juniper

**Affiliations:** 1 Department of Animal Sciences, School of Agriculture, Policy and Development, University of Reading, P.O. Box 237, Earley Gate, Reading RG6 6EU, UK; superqnb100@sina.com (N.Q.); fg910722@gmail.com (G.F.); s.a.beauclercq@reading.ac.uk (S.B.); joepaxtonpitt@yahoo.co.uk (J.P.); eric.newton@pgr.reading.ac.uk (E.E.N.); A.Angelidis@pgr.reading.ac.uk (A.E.A.); d.t.juniper@outlook.com (D.T.J.); 2 Georgikon Campus, Szent Istvan University, Deák Ferenc u. 16, H-8360 Keszthely, Hungary; 3 Matís Ltd., Vinlandsleid 12, 113 Reykjavik, Iceland; natasa@matis.is (N.D.); asta.h.petursdottir@matis.is (Á.H.P.); helgag@hi.is (H.G.); 4 Institute for Food, Nutrition and Health, University of Reading, P.O. Box 237, Earley Gate, Reading RG6 6EU, UK; d.i.givens@reading.ac.uk; 5 Centre for Dairy Research, School of Agriculture, Policy and Development, University of Reading, Hall Farm House, Church Ln, Reading RG2 9HX, UK; d.j.humphries@reading.ac.uk; 6 Faculty of Food Science and Nutrition, School of Health Sciences, University Iceland, 102 Reykjavik, Iceland

**Keywords:** dairy cow, grazing, heavy metals, macrominerals, milk, organic, pasture, production system, trace elements

## Abstract

Thirty conventional and twenty-four organic dairy farms were divided into equal numbers within system groups: high-pasture, standard-pasture, and low-pasture groups. Milk samples were collected monthly for 12 consecutive months. Milk from high-pasture organic farms contained less fat and protein than standard- and low-pasture organic farms, but more lactose than low-pasture organic farms. Grazing, concentrate feed intake and the contribution of non-Holstein breeds were the key drivers for these changes. Milk Ca and P concentrations were lower in standard-pasture conventional farms than the other conventional groups. Milk from low-pasture organic farms contained less Ca than high- and standard-pasture organic farms, while high-pasture organic farms produced milk with the highest Sn concentration. Differences in mineral concentrations were driven by the contribution of non-Holstein breeds, feeding practices, and grazing activity; but due to their relatively low numerical differences between groups, the subsequent impact on consumers’ dietary mineral intakes would be minor.

## 1. Introduction

Cow milk is a common component of human diets in many areas of the world due to its considerable nutritional value, specifically as an important source of several key minerals that support various human biochemical processes by serving structural and functional roles [1,2,3]. It has been shown to provide approximately 21% of Ca, 26% of I, 7% of Mg and 6% of K relative to an adult’s daily requirement (UK data; [4]), playing positive roles in the health of bones and teeth, muscle contraction, cells, membrane structure, energy metabolism, LDL-responses to high-fat dairy intake, hypertension, certain cancers, obesity, kidney stones, metabolic rate, thermoregulation, growth and reproduction, atherosclerosis, and oxidative stress [2,3,4]. In addition, mineral concentrations in milk, as well as their distribution in the micellar/serum phase, affects cheese-making properties in milk such as coagulation, buffering, curd firmness and overall quality, clotting time, protein and fat recovery, and the weight of whey [5]. For example, Ca may increase the retention of water in the curd, reduce clotting time, improve curd firmness, increase protein and fat recovery, and benefit the coagulation pattern and cheese-making properties; P may increase solids content in the curd, but excessive P may reduce fat recovery; high Na concentrations may reduce protein recovery, while increased contents of Mg may result in slower coagulation and lower cheese yields [5,6].

Recently, there has been an increasing preference of consumers for dairy products produced from pasture-based systems (either organic or pasture-based non-organic) [7]. Previous work has shown that increased pasture intake of cows, which may also be associated with changes in other husbandry practices (concentrate feed intake, breed, housing, etc.), influences a range of milk quality attributes, including basic composition, fatty acids, protein, carotenoids, antioxidants and mineral profiles [8,9,10,11]. For example, higher pasture intake (typically associated with lower intakes of concentrate feed) has been linked to higher concentrations of polyunsaturated fatty acids (including the nutritionally-beneficial omega-3), in previous farm surveys, and, in some occasions, with lower concentrations of nutritionally undesirable saturated fats (including palmitic acid) [12,13]. These results have been also observed in several studies at retail level where milk produced from typically higher-grazing systems (organic) or seasons (spring/summer) was compared with milk from lower-grazing conditions (conventional systems and/or during autumn/winter) [14,15,16], as well as in recent meta-analyses [17]. Production systems which rely more on grazing (low-input, organic, outdoors) have resulted in milk with higher concentrations of the antioxidants retinol, xanthophyll, lutein, zeaxanthin, β-cryptoxanthin, α-carotene, β-carotene, and total carotenoids [12,13,18], as well as the protein β-lactoglobulin [12,13,19]. Although the contribution of alternative breeds (non-Holstein) to the herd has been found to be a significant driver for the concentrations of fatty acids, tocopherols, antioxidants and minerals in milk, the relative impact of animal diet (in particular pasture intake) on these parameters has been more pronounced than breed, based on previous multivariate redundancy analyses [12,20]. In contrast, breed was seen to be similarly important, and in some cases stronger of a, driver for milk protein content and profile [12,13].

Previous retail-based and farm-based studies have shown differences in mineral concentrations between organic and conventional milk [20,21], while also mentioning that differences in pasture intake might be a key driver for these findings. Organic milk was reported to contain more Ca, P, K and Mo, and less Cu, Fe, I, Mn and Zn when compared with conventional milk [20,21]. Although differences in milk mineral profiles between organic and conventional milk has been attributed mainly to different grazing management between the two production systems, which includes intakes of pasture, concentrate and other components of smaller weights in the diet [20,22], in practice the terminology ‘organic’ and ‘conventional’ can be broad and great variation in husbandry practices also exists within both production systems, as previously illustrated in farm surveys [12,13].

Given that within-system variation in husbandry practices recorded in previous farm surveys [12,13] is known to affect mineral intakes of cows, and therefore milk mineral profiles [20,22,23], within-system differences in milk mineral concentrations may also exist. The present study therefore aimed to (i) investigate the effects of grazing management within organic and conventional dairy systems, with emphasis on pasture intake, on milk production, composition and mineral and heavy metal profiles; (ii) identify and quantify the relative impacts of different husbandry practices (feeding, breeding, management) on the same parameters; and (iii) estimate the impact on consumers’ mineral intakes when milk produced under different grazing management is consumed.

## 2. Materials and Methods

### 2.1. Design of the Survey and Experiment

Milk samples were collected monthly between January and December 2019 from the bulk tank on the farms after stirring, from 54 dairy herds in Southern England. In total, 359 milk samples were collected from 30 conventional farms and 283 milk samples were collected from 24 organic farms certified according to the standards of Soil Association or Organic Farmers and Growers; collection of six samples was missed throughout the survey. Records related to herd breeding (contribution of different breeds, and crossbreed cows in the herd) and feeding (different types and amounts of offered conserved forages, other feeds, supplements) were collected via questionnaires, which were completed by dairy producers and an interviewer. Average liveweights for each breed (Supplementary Material Table S1) and the contribution of the different breeds in each herd were used to estimate herd average liveweight [24]. The latter, together with the corresponding milk yield, were used to calculate total dry matter intake (DMI), while pasture intake was calculated as the difference between calculated DMI and the measured offered DM (conserved forages, other feeds, and supplements) [19]. In both conventional and organic production systems, all cows were provided with access to the outdoors. The 30 conventional farms were split into three experimental groups (10 farms each) to represent different grazing management in the farms with respect to the average contribution of pasture to the diet during the grazing season (April–September), including a high-pasture group (CHP; 28–65% DMI), a standard-pasture group (CSP; 5–18% DMI), and an outdoors with low/limited pasture group (CLP; 0–3% DMI). The 24 organic farms were similarly split into three experimental groups (8 farms each) including a high-pasture group (OHP; 60–74% DMI), a standard-pasture group (OSP; 39–59% DMI), and a low-pasture group (OLP; 13–34% DMI).

### 2.2. Analysis of Milk Samples for Basic Composition and Minerals

Detailed procedures of milk analysis were described in Qin et al. [20]. Briefly, contents of milk fat, protein and lactose were quantified using Fourier transform infrared spectroscopy (MilkoScanTM 7RM; FOSS, Hillerød, Denmark), and SCC was determined by flow cytometry (FossomaticTM 7; FOSS, Hillerød, Denmark), both in commercial laboratories (National Milk Laboratories, Wolverhampton, UK). Concentrations of milk minerals and heavy metals, including aluminium (Al), arsenic (As), calcium (Ca), cadmium (Cd), cobalt (Co), chromium (Cr), copper (Cu), iron (Fe), mercury (Hg), iodine (I), potassium (K), magnesium (Mg), manganese (Mn), molybdenum (Mo), sodium (Na), nickel (Ni), phosphorus (P), lead (Pb), selenium (Se), tin (Sn), and zinc (Zn), were analysed by inductively coupled plasma mass spectrometry (Agilent 7900, Agilent Technologies, Singapore) in Matis (Reykjavik, Iceland). Milk I concentrations were analysed based on previously published methods [22]. Analyses of the remaining elements were performed according to NMKL 186 [25] (accreditation: IST/ISO 17025/2005, SWEDAC).

### 2.3. Statistical Analysis

Mixed linear models (residual maximum likelihood analysis; REML; [26]) were used for the analysis of variance in GenStat (VSN International, 18th Edition, Hempstead, UK). The two databases, representing conventional and organic production systems, were analysed separately. In mixed linear models, the fixed effects included pasture intake (CHP, CSP and CLP for conventional production system; OHP, OSP and OLP for organic production system), month (January–December), and their interaction; and the random effect was Farm ID. The main effects were considered to have a significant effect when *p* < 0.05; tendencies towards significant effect were considered at 0.05 ≤ *p* < 0.10. Normality plots of the residuals of the final model were assessed and, as a result, the mineral concentration parameters and SCC transformed before REML analysis (log(x + 1) and log(x), respectively). Means and standard errors were calculated using the untransformed data. Fisher’s least significant difference test was used for pairwise comparisons where the effect of a fixed factor, or their interaction, was significant.

Multivariate redundancy analysis (RDA), in CANOCO 5.12 Windows Release, were carried out in order to evaluate the relative impact of breed and diet parameters on milk yield, basic composition and mineral profile [27], with Monte Carlo permutation tests in order to perform the automatic forward selection of variables and calculate significant effects. In the RDA biplots, the arrows’ length and direction depict the relative impact of herd breed and diet composition parameters on milk yield, basic composition, and mineral concentration parameters. The two databases, representing data from conventional and organic production systems, were analysed separately. In both RDA, the breed driver represents the contribution of non-Holstein breeds in the total herd. Drivers related to diet represent the proportions of individual feeds in the total diet (as % DMI); while supplementation of minerals and vitamins was included as g/day. The response variables were parameters related to milk productivity, milk basic composition, and mineral profiles.

## 3. Results

### 3.1. Breeding and Feeding in the Herds

Breed composition and intakes of feed in conventional and organic herds are shown in Table 1. Monthly intakes of dry matter, total forage, total concentrate, grazing, grass/clover silage, and maize silage in different groups are presented as Supplementary Materials (Figure S1 for conventional farms and Figure S2 for organic farms). In conventional herds, Holstein represented more than 63% of the total herd across all groups. The CHP farms tended to have a lower (*p* = 0.08) contribution of Holstein in the herds when compared with the CSP (−25.3% herd) and CLP (−32.6% herd) farms. Instead, the CHP farms had more (*p* < 0.05) cows of other breeds/crossbreds, which were not in the other two groups. The proportions of other breeds in the herds were small and they were similar between groups. In organic herds, Holstein was also the most abundant breed, comprising more than 40% of the total herd in all groups. The contribution of Holstein tended to differ between groups (*p* = 0.07), as a higher number was involved in the OLP farms than the OHP (+47.1% herd) and OSP (+27.5% herd) farms. The OLP farms had no Ayrshire cows, although Ayrshire comprised 13% of the herds in OHP and OSP farms. Other breeds/crossbreds were minimal in the OLP herds (−37.7% herd vs. OHP; −20.3% herd vs. OSP). Differences in the contribution of all other breeds were less than 5%.

Grazing management had significant effects on intakes of DM (*p* = 0.02), forage (*p* < 0.001), concentrate (*p* < 0.001), grazing (*p* < 0.001), maize silage (*p* = 0.01) and oils (*p* = 0.01) in conventional farms (Table 1). Total forage and pasture intakes differed (*p* < 0.05) between all groups, increasing with the rise of pasture intake (CHP > CSP > CLP), while the opposite (*p* < 0.05) was observed for total concentrate intake (CHP < CSP < CLP). Intakes of DM, maize silage and oils were lower (*p* < 0.05) in the CHP group when compared with the other conventional farm groups. Mineral intake in conventional farms had a tendency towards significant difference between groups (*p* = 0.06); the intakes in the CSP and CLP groups were 1.8- and 2.4-fold higher compared to that of the CHP group.

In organic production system, grazing management affected intakes of DM (*p* = 0.02), forage (*p* < 0.001), concentrate (*p* < 0.001), grazing (*p* < 0.001) and wholecrop silage (*p* = 0.04; Table 1). Intakes of DM and wholecrop silage were higher (*p* < 0.05) in the OLP group when compared with the OHP group, whereas these parameters in the OSP group were similar as those in the OLP and OHP groups. The OLP group had a lower (*p* < 0.05) total forage intake when compared with the other two organic farm groups, while the opposite (*p* < 0.05) was observed in total concentrate intake. Pasture intake differed (*p* < 0.05) between all organic farm groups (OHP > OSP > OLP). Mineral intake tended to differ (*p* = 0.06) between groups, as the OLP group’s intake was 1-fold greater than those of the other two organic farm groups.

### 3.2. Milk Yield and Basic Composition

**Figure 1:**
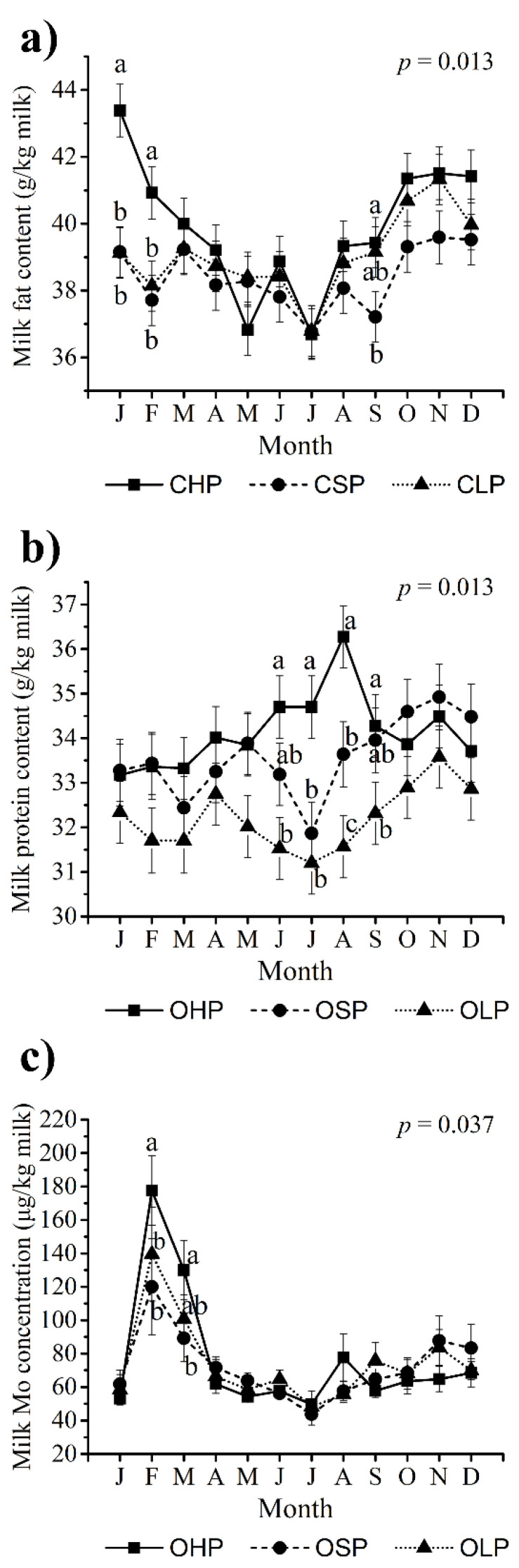
Concentrations of milk components and minerals where a significant interaction between pasture intake and month was observed. (**a**) Milk fat content in the conventional farms; (**b**) Milk protein content in the organic farms; (**c**) Milk Mo concentration in the organic farms. In (**a**,**b**), predicted means from the fitted mixed linear model are shown. In (**c**), means of the measured values are shown, while the *p*-values were obtained from the fitted mixed linear model based on the log(x + 1) transformed value. The error bars depict the means’ standard errors. Significant differences between groups within months are indicated with different letters (*p* < 0.05). CHP, conventional high-pasture feeding farms; CSP, conventional standard-pasture feeding farms; CLP, conventional low-pasture feeding farms; OHP, organic high-pasture feeding farms; OSP, organic standard-pasture feeding farms; OLP, organic low-pasture feeding farms.

In the conventional production system, the CHP group had a lower (*p* < 0.05) average daily milk yield per cow when compared with the CSP (−5.4 kg/d) and CLP (−2.1 kg/d) groups (Table 2). Average contents of milk fat, protein, lactose, and SCC were not affected by grazing management in conventional farms. Across groups with different pasture intakes, variation in milk yield and contents of fat, protein, lactose, and SCC over months was observed (Supplementary Table S2). A significant (*p* = 0.01) interaction between pasture intake and month was observed in the fat content of conventional milk (Figure 1). The comparisons within months suggested a higher (*p* < 0.05) milk fat content in the CHP group in January and February when compared with the other two groups, and in September when compared with the CSP group. In general, the milk fat content was highest in winter and lowest in summer across all groups (Figure 1a).

In the organic production system, the OLP group had a higher (*p* < 0.05) average daily milk yield per cow than the OSP (+3.9 kg/d) and OHP (+6.0 kg/d) groups, but lower (*p* < 0.05) average milk fat and protein contents when compared with the other two groups (Table 2). Milk lactose content was higher (*p* < 0.05) in the OLP group than the OHP group. Milk SCC was not affected by pasture intake in the organic production system. Regardless of pasture intake, concentrations of milk fat (*p* < 0.001), protein (*p* = 0.002), lactose (*p* < 0.001) and SCC (*p* < 0.001) in the organic production system varied over months (Supplementary Table S2). The protein content of organic milk showed a significant (*p* = 0.01) interaction between pasture intake and month (Figure 1b). The OHP group had a higher (*p* < 0.05) milk protein content (i) in June and September when compared with the OLP group, and (ii) in July and August when compared with the other two groups. Moreover, milk protein content in the OSP group was higher (*p* < 0.05) than that in the OLP group in August.

### 3.3. Milk Mineral Profile

In conventional production systems, grazing management influenced average concentrations of Ca (*p* = 0.001) and P (*p* = 0.045; Table 2). Milk Ca and P concentrations were lower (*p* < 0.05) in the CSP group when compared with the CHP (−49.5 mg/kg milk Ca; −24.9 mg/kg milk P) and CLP (−44.6 mg/kg milk Ca; −29.0 mg/kg milk P) groups. Concentrations of other analysed macrominerals, including K, Mg, and Na, did not differ between conventional farm groups. Of the analysed essential trace elements, concentrations of Cu, Fe, I, Mn, Mo, and Zn in conventional milk revealed no effect of pasture intake (Table 2). Concentrations of Co and Se were very low; as the majority of individual measurements of Co (74%) and Se (83%) were below the limits of quantification (LOQ; 0.59 μg/kg milk fo Co; 35.38 μg/kg milk for Se), they were excluded from statistical analyses. The same was observed for the concentrations of most non-essential trace elements and toxic heavy metals (As, Cd, Cr, Hg, Ni, and Pb), and similarly they were excluded from the statistical analyses. The LOQs were: 5.90 μg/kg milk for As; 0.24 μg/kg milk for Cd; 7.08 μg/kg milk for Cr; 2.36 μg/kg milk for Hg; 4.72 μg/kg milk for Ni; 8.26 μg/kg milk for Pb. The proportions of individual measurements which were below LOQs were: As, 97%; Cd, 56%; Cr, 77%; Hg, 99%; Ni, 84%; Pb 97%. With respect to the accurately quantified non-essential trace elements, concentrations of Al and Sn in conventional milk were not influenced by pasture intake (Table 2). Regardless of pasture intake, concentrations of all analysed elements had significant (*p* < 0.05) variation over months, and the monthly data is presented in supplementary Table S3. The scatter plots of all measurements of mineral concentrations in conventional herds are presented in supplementary Figure S3.

In organic production systems, grazing management affected milk Ca concentration (*p* = 0.008) and tended to affect concentrations of Mg (*p* = 0.07) and Na (*p* = 0.07; Table 2). Milk Ca concentration was lower (*p* < 0.05) in the OLP group than the OHP (−44.3 mg/kg milk) and OSP (−36.6 mg/kg milk) groups. Moreover, the OLP group had numerically lower milk Mg and Na concentrations when compared with the OHP (−3.9 mg/kg milk Mg; −28.5 mg/kg milk Na) and OSP (−2.2 mg/kg milk Mg; −15.3 mg/kg milk Na) groups. Similar to conventional production systems, results of Co and Se concentrations in organic milk were excluded from the statistical analyses because 81% and 80% of their individual measurements were below LOQs, respectively. Concentrations of Cu, Fe, I, Mn, Mo, and Zn in organic milk were not affected by grazing management (Table 2). However, Mo concentration showed a significant (*p* = 0.04) interaction between pasture intake and month, as characterized by a higher (*p* < 0.05) concentration in the OHP group in February when compared with the other two groups and in March when compared with the OSP group (Figure 1c). Of the analysed non-essential trace elements and toxic heavy metals, As, Cd, Cr, Hg, Ni, and Pb were present at low concentrations in organic milk as well. The proportions of individual measurements which were below LOQs were: 98% for As; 63% for Cd; 82% for Cr; 100% for Hg; 89% for Ni; 96% for Pb. Of the accurately quantified non-essential trace elements, Al concentration in organic milk did not differ between groups (Table 2). In contrast, milk Sn concentration was higher (*p* < 0.05) in the OHP group when compared with the OSP (+1.65 μg/kg milk) and OLP (+2.33 μg/kg milk) groups. Across groups, concentrations of all of the analysed elements had significant (*p* < 0.05) variation over months, and the monthly data is presented in supplementary Table S4. The scatter plots of all measurements of mineral concentrations in organic herds are presented in supplementary Figure S4.

### 3.4. Effect of Breeding and Diet Parameters

**Figure 2:**
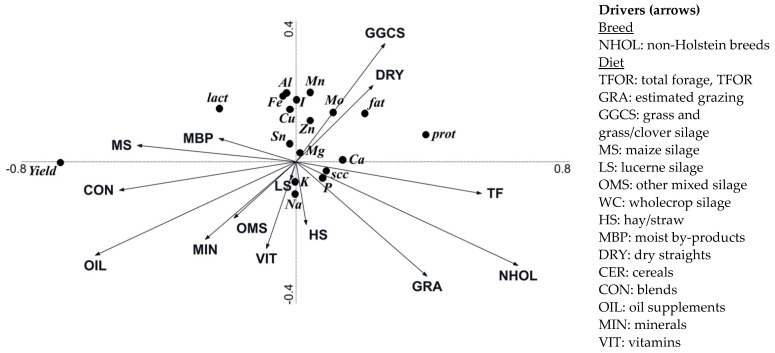
Biplots of the conventional farm data (as analysed by multivariate redundancy analysis) illustrating relationships between breeding parameters (NHOL) and diet composition parameters (TFOR, GRA, GGCS, MS, LS, OMS, WC, HS, MBP, DRY, CER, CON, OIL, MIN, VIT) relative to the (i) milk yield (*Yield*) and contents of fat (*fat*), protein (*prot*), lactose (*lact*), somatic cell count (*scc*), aluminium (*Al*), calcium (*Ca*), copper (*Cu*), iron (*Fe*), iodine (*I*), potassium (*K*), magnesium (*Mg*), manganese (*Mn*), molybdenum (*Mo*), sodium (*Na*), phosphorus (*P*), tin (*Sn*), and zinc (*Zn*). Axis 1 explained 49.4% of the variation, and Axis 2 an additional 0.1%. The *p*-values for the drivers that contributed to the explained variation was as follows: NHOL (*p* = 0.002), MS (*p* = 0.002), CON (*p* = 0.002), GGCS (*p* = 0.002), OIL (*p* = 0.002), LS (*p* = 0.002), TF (*p* = 0.006), OMS (*p* = 0.016), HS (*p* = 0.018), MBP (*p* = 0.036), VIT (*p* = 0.038), MIN (*p* = 0.138), DRY (*p* = 0.272), GRA (*p* = 0.696).

The RDA for conventional herd data assessed the relationships between drivers related to cow breeding and diet composition with milk yield, basic composition, and contents of minerals (Figure 2). The drivers explained 49.6% of the variation, of which 49.5% was explained by axis 1 and a further 0.1% was explained by axis 2. Non-Holstein breeds, and intakes of maize silage and blends were the most influential parameters, explaining 22.0%, 9.5% and 6.7% of the variation, respectively; followed by intakes of grass/grass-clover silage (2.9%), oil (1.9%), lucerne silage (1.6%), and total forage (1.3%). Other feeds individually accounted for less than 1% of the total explained variation. Milk yield was positively associated with intakes of maize silage, blends, oil, moist by-products and minerals, and negatively associated with non-Holstein breeds, and intakes of pasture and total forage. Milk lactose contents were negatively associated with non-Holstein breeds and intakes of total forage and pasture, and positively associated with intakes of maize silage, moist by-products, and blends. Concentrations of Al, Fe, and I in milk were positively associated with intakes of grass/grass-clover silage and dry-straights, and negatively associated with non-Holstein breeds and intakes of pasture, hay/straw, other mixed silages, and vitamins. Concentrations of fat, protein, Mn, Cu, Mo and Zn in milk were positively associated with intakes of grass/grass-clover silage and dry-straights, and negatively associated with intakes of blends, oil, minerals, vitamins, hay/straw and other mixed silages. SCC and concentrations of Ca and P in milk were positively associated with non-Holstein breeds and intakes of total forage and pasture, and negatively associated with intakes of blends, maize silage, moist by products, blends, and oil. Na concentrations in milk were positively associated with non-Holstein breeds and intakes of pasture, hay/straw, other mixed silages, and vitamins, and negatively associated with intakes of grass/grass-clover silage and dry-straights.

**Figure 3:**
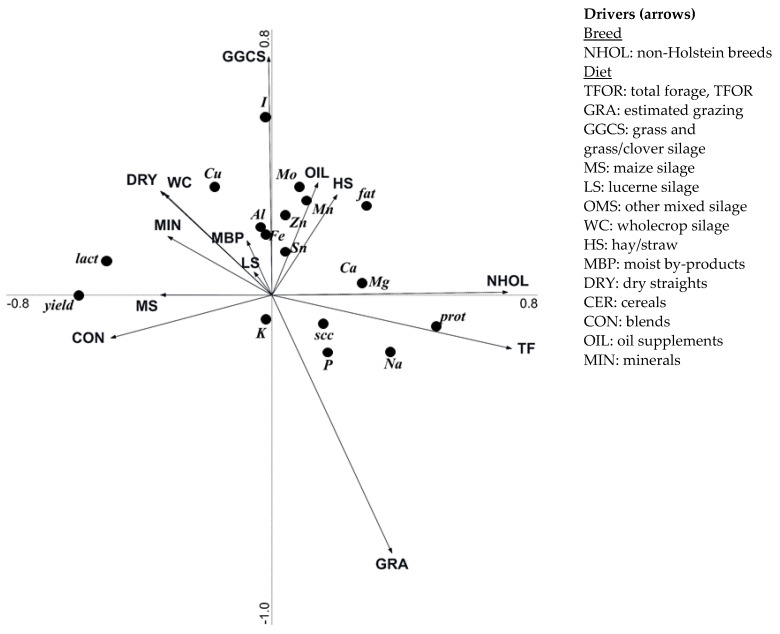
Biplots of the organic farm data (as analysed by multivariate redundancy analysis) illustrating relationships between breeding parameters (NHOL) and diet composition parameters (TFOR, GRA, GGCS, MS, LS, OMS, WC, HS, MBP, DRY, CER, CON, OIL, MIN) parameters relative to the (i) milk yield (*Yield*) and contents of fat (*fat*), protein (*prot*), lactose (*lact*), somatic cell count (*scc*), aluminium (*Al*), calcium (*Ca*), copper (*Cu*), iron (*Fe*), iodine (*I*), potassium (*K*), magnesium (*Mg*), manganese (*Mn*), molybdenum (*Mo*), sodium (*Na*), phosphorus (*P*), tin (*Sn*), and zinc (*Zn*). Axis 1 explained 37.2% of the variation, and Axis 2 an additional 0.3%. The *p*-values for the drivers that contributed to the explained variation was as follows: TF (*p* = 0.002), NHOL (*p* = 0.002), MS (*p* = 0.032), MIN (*p* = 0.062), GGCS (*p* = 0.064), DRY (*p* = 0.158), MBP (*p* = 0.190), HS (*p* = 0.216), GRA (*p* = 0.236), OIL (*p* = 0.332), LS (*p* = 0.708), CON (*p* = 0.738), WC (*p* = 0.788).

RDAs for organic herd data examined relationships between drivers related to cow breeding and diet composition with milk yield, basic composition, and contents of minerals (Figure 3). Drivers explained 37.5% of the variation, of which 37.2% was explained by Axis 1 and a further 0.3% was explained by Axis 2. Intakes of total forage and non-Holstein breeds were the most influential parameters, explaining 20.8% and 12.1% of the variation, respectively; followed by maize silage (1.1%). Other feeds individually accounted for less than 1% of the total explained variation. Milk yield and lactose concentrations were positively associated with intakes of maize silage, blends, dry-straights, wholecrop and minerals, and negatively associated with non-Holstein breeds, and intakes of total forage and pasture. Milk concentrations of I, Cu, Al, and Fe were negatively associated with intakes of pasture and total forage, and positively associated with intakes of grass/grass-clover silage, dry-straights, wholecrop and minerals. Concentrations of milk fat, Mo, Mn, Zn and Sn were positively associated with non-Holstein breeds and intakes of grass/grass-clover silage, oils and hay/straw, and negatively associated with intakes of maize silage and blends. Concentrations of milk Ca, Mg were positively associated with non-Holstein breeds and intakes of total forage and pasture, and negatively associated with intakes of maize silage, and blends. Milk SCC and concentrations of protein, Na and P were positively associated with non-Holstein breeds and intakes of total forage and pasture, and negatively associated with intakes of maize silage, blends, dry-straights, wholecrop, and minerals. Milk K concentrations were positively associated to pasture intake, and negatively associated to intakes of grass/grass-clover silage, oil and hay/straw.

## 4. Discussion

### 4.1. Milk Yield and Basic Composition

Daily milk yield per cow was lower in the CHP than the CSP and CLP groups, and lower in OHP and OSP groups than OLP. A pattern where milk yield decreased with increasing pasture intake can be seen in both production systems. As reflected by feed intake records, pasture in the diets was increased at the expense of concentrate and maize silage. Such changes, in particular the reduction of concentrate, can decrease the energy density of diets and the energy supply for milk production [12,28]. This was supported by the RDA results that milk yield was positively associated with intakes of blends and maize silage in both production systems, while it was negatively associated with total forage intake. In addition, RDA results showed that milk yield was negatively associated with the contribution of non-Holstein breeds in the herd. Previous findings have suggested a higher milk yield associated with Holsteins when compared with alternative breeds, such as Jersey and Brown Swiss [28,29]. Interestingly, in both production systems in the present study, farms that fed cows a higher level of pasture also had a higher contribution of non-Holstein breeds in the herd. The reason for this pattern might be that some alternative breeds to Holstein adapt better to grazing, in terms of the demands of high concentrate intake and veterinary input [30], and thus farmers that chose to introduce more pasture in cow diets would also form herds with higher contribution of alternative breeds. The difference in concentrate intake and herd breed composition may have jointly resulted in the difference in milk yield between farm groups.

Farm grazing management consistently affected the contents of milk components in organic but not conventional production systems. In organic production systems, the average milk fat content was lower in OLP farms than in OHP and OSP farms. Although the average milk fat content did not differ between conventional farm groups, similar patterns were observed in specific months, as characterized by the higher fat contents in the CHP group in January and February when compared with both CSP and CLP groups, and in September when compared with the CLP group. These results are contradictory to the findings of Couvreur et al. [31], who observed a linear decrease in milk fat content of Holstein cows with the increasing replacement of maize silage by fresh grass in the diet. However, dietary pasture in the present study was increased at the expense of concentrate (in particular cereals and blends) and wholecrop silage, which may explain these results. Differences in concentrate intake and types between organic and conventional farms contributes to variation in milk fat content because cereal grains affect ruminal biohydrogenation by increasing the production of specific trans fatty acids that inhibit milk fat synthesis [32]. The results of the present study are in line with previous findings showing that cows managed in a pasture-based system produce milk with higher fat contents when compared with those managed in a total mixed ration (TMR) feeding system without fresh pasture intake [10,33]. Moreover, Walker et al. [34] fed cows different levels of concentrate and observed a linear decrease of milk fat content when concentrate intake was increased from 0% DM to 40% DM. In the present study, cows in the OLP farms consumed a higher amount of concentrate (31% DM) than their counterparts in other organic farm groups (22.3% DM and 23.5% DM), therefore being a potential factor that reduced milk fat content. The same explanation applies to conventional production systems where lower amounts of concentrate were fed in the CHP group in the months when the CHP group had higher milk fat content than CSP and/or CLP groups. This was supported by RDA results that milk fat content was negatively associated with the intake of blends in both production systems. In addition, the different milk fat contents between organic farms of different grazing management were probably due to the considerably higher proportions of alternative breeds in OHP and OSP (40.2% and 59.8% of the herd) groups than OLP (87.3%). Some alternative breeds, such as Ayrshire, Jersey and Guernsey, produce milk with a higher fat content when compared with Holstein [35]. Therefore, the higher proportions of non-Holstein breeds may have also contributed to the higher milk fat contents in the OHP and OSP farms, as also supported by the positive correlation between milk fat content and the contribution of non-Holstein breeds identified in the RDA analysis.

Milk protein concentration is generally positively associated with dietary energy and protein intake [36]. However, a lower average milk protein content was observed in the OLP group, in which cows likely had higher intakes of energy and protein due to the increased intakes of DM and concentrate, when compared with OHP and OSP farms. These results correspond to a previous finding that a pasture-based system produced milk with higher protein concentration than a TMR feeding system without pasture feeding [33]. Moreover, similar results were reported by Couvreur et al. [31] that milk protein concentration increased linearly with increasing proportions of fresh grass in Holstein cows’ diets, and they suggested that the increase in protein content was caused by increased propionic acid production in the rumen that modified energy supply to the mammary gland. The effect of pasture was also reflected by the significant interaction between pasture intake and month in milk protein content in the present study, showing that the difference between organic farm groups was maximized between June and September, during which the pasture intake of all groups and the difference in pasture intake between groups have also reached the maximum. The RDA results revealed that protein content was strongly positively associated with total forage intake, and negatively with the intake of blends. Pasture intake was also positively associated to milk protein content, but to a lesser extent. In addition, protein content was strongly positively associated with the contribution of non-Holstein breeds, in line with the previous report about the variation in milk protein content between cow breeds [29,37]; therefore, breed differences also contributed to this observation.

Milk lactose content decreased with increasing pasture intake in organic production systems, and a significant difference was observed between OHP and OLP farms. However, herd breed composition might be the primary factor influencing milk lactose content, considering the strong negative correlation between milk lactose content and the contribution of non-Holstein breeds in the RDA results and the previously demonstrated difference in milk lactose content between Holstein, Jersey, and Brown Swiss [29]. Dietary factors may have also contributed to the difference in milk lactose content between organic farm groups, as lactose content was positively associated with intakes of blends, maize silage and wholecrop, and negatively associated with total forage intake. A previous study reported that milk lactose concentration was increased by abomasal infusion of starch to cows [38], which suggests that increasing cows’ intake of cereal grain may increase milk lactose content. Therefore, higher milk lactose content in the OLP than OHP farms may have also resulted from the higher contribution of concentrate in the cows’ diet, in particular from cereal-based blends.

The current study highlights that there are husbandry practices that influence both organic and conventional systems similarly, while the effect of other practices may depend on the system. According to the RDA results, conventional and organic production systems shared some similar correlations between the concentrations of milk components and herd and dietary factors. For instance, in both production systems, fat and protein contents were positively associated with total forage intake, while they were negatively associated with blend intake; additionally, lactose content was negatively associated with the contribution of non-Holstein breeds. However, differences in milk composition between farm groups were less significant in conventional production systems when compared with organic production systems. The main reason for the difference might be that organic farms were consisted overall of more cows from alternative breeds to Holstein than conventional farms (12.7–60.8% vs. 3.6–37.2%) to achieve acceptable robustness, longevity and productivity of animals [39]. The overall higher contribution of non-Holstein breeds and the greater breed differences between farm groups might have produced greater differences in milk composition in organic production systems when compared with conventional production systems. This was reflected by the RDA results that showed the correlations of milk fat and protein contents with the contribution of non-Holstein breeds in conventional production systems were less significant than those in organic production systems.

### 4.2. Milk Mineral Profile

Milk Ca concentration was higher in organic farms with higher pasture intake. Interestingly, in conventional production systems, Ca concentration was lower in the standard-pasture farms (CSP) when compared with those feeding from the high (CHP) or low (CLP) levels of pasture, and so was milk P concentration. The concentrations of Ca and P in milk are relatively constant and are mainly determined by genetics [1,37]. Therefore, the differences in Ca and P concentrations between farm groups were likely associated with the different breed make-ups of the herds. RDA results of conventional production systems in the present study showed that milk Ca and P concentrations were positively associated with the contribution of non-Holstein breeds. Compared with CHP and CLP farms (2.8% and 1.7%, respectively), CSP farms (10.0%) had more British Friesian in the herds, being a potential explanation for the lower milk Ca and P concentrations in these farms. In organic production systems, the contribution of non-Holstein breeds was also positively associated with milk Ca concentration, while it was less associated with P concentration.

More than 65% of milk Ca is associated with casein micelles, while 50% of the inorganic phosphate is also found in milk solid fraction [1,40]. In organic systems, protein concentrations were higher in OHS and OSP groups, thus being in line with the higher concentrations of Ca in the milk from the same herds, while milk P content in these groups was also numerically higher when compared with the OLP group, although the difference was not statistically significant. In conventional systems, although not statistically significant, the CSP group had numerically lower milk fat and protein contents, and this may have been also reflected in milk Ca and P contents. Casein micelles may contain about one-third of the Mg in milk [40], and although there was only a tendency (*p* = 0.069) for an effect of grazing management on milk Mg concentrations in the organic herds, OHP and OSP had numerically higher Mg concentrations than OLP. These results demonstrate that in addition to the effect of breed, dietary factors that influence milk fat and protein contents described above, including intakes of blends, pasture and total forage, may also play an indirect role on milk concentrations of Ca and P, and potentially Mg. The RDA demonstrated that Ca and P in milk were positively associated with intakes of total forage and pasture, and negatively associated with intakes of blends, maize silage, moist by-products, oil and/or wholecrop.

All of the analysed essential trace elements had similar average concentrations in all farm groups in both production systems. Concentrations of essential trace elements in milk are affected by animals’ concentrate intake because of the routine supplementation of these elements to concentrate feeds [11]. In the present study, cows’ total concentrate intake declined with increasing pasture intake in both production systems, and so did the intake of minerals numerically, which were likely supplemented to concentrates as a routine. However, the difference in concentrate and mineral intakes between farm groups may have not been sufficient to produce different concentrations of milk essential trace elements. Most of the essential trace elements clustered in the RDA plots (e.g., Fe, Cu, I, Mn, Mo and Zn), thus revealing common patterns of correlation between their concentrations in milk and dietary and herd factors. In the conventional production system, their concentrations were positively associated with intakes of grass/grass-clover silage and dry straights, and negatively associated with intakes of grazing, minerals, vitamins, and other mixed silages. In the organic production system, their concentrations were positively associated with intakes of grass/grass-clover silage, oil and hay/straw, and negatively associated with pasture intake.

Most of the analysed non-essential trace and heavy metal elements were present in very low concentrations in milk. Of the accurately quantified ones, milk Sn concentration was higher in OHP than OSP and OLP farms. A similar numerical difference was observed in conventional milk Sn concentration as well, although the difference between farm groups was not statistically significant. Previous literature about milk Sn concentration and its influencing factors is scarce. Zwierzchowski and Ametaj [41] suggested that non-essential trace and heavy metal elements in milk are primarily originated from soil or contaminated water. Therefore, the higher milk Sn concentration in the high-pasture farms could be potentially related to the greater grazing activity when compared with the other farm groups, as soil ingestion may be increased when grass is grazed compared with feeds delivered indoors to a feeder [11]. Heavy metal concentrations in milk were only traces, mostly below an already extremely low LOQ, reinforcing that it cannot be considered a source of heavy metals for humans as the measured traces were largely lower than the maximum recommendations for milk; therefore, there is no association with any health impacts.

Interestingly, although the RDA showed that pasture intake is a strong driver for milk mineral concentrations in both production systems, grazing management practices within-conventional and within-organic farms did not seem to greatly affect milk mineral concentrations beyond (low-moderate) differences in Ca and/or P. This may reveal that although grazing has the potential to affect milk mineral concentrations, within-system pasture intake differences such as the ones explored in this study (0–26% DMI for conventional herds, and 14–37% for the organic herds) may not be strong enough to demonstrate this potential. However, based on the RDA information from the present study, it may not be surprising if systems with higher contrast in pasture intakes (e.g., low-input vs. high-intensive [12,13]) result in milk with more extensive differences in their mineral concentrations; this is something that future studies could investigate.

### 4.3. Potential Impact of Grazing Management on the Mineral Intakes of UK Consumers

Milk is an important source of many minerals in human diets, including Ca, I, K, Mg and Zn [4]. Given that milk mineral profiles can be affected by farm grazing management, daily mineral intakes of consumers may be also impacted. An assessment of the impact of farm grazing management on consumers’ mineral intake from milk was conducted in the present study. The intakes of liquid milk in the UK population (covering different demographics in terms of gender and age) were calculated using total energy intake data from the National Diet and Nutrition Survey [4] and the average energy concentration of whole milk [42]. The population intakes of elements that were significantly affected by grazing management (Ca, P and Sn) were calculated using the estimated milk intakes (for the different demographics) and milk concentrations, as quantified in the present study; following that, a comparison of the calculated intakes with the reference nutrient intakes (RNI) was performed [4].

Depending on the intakes in the UK population, the nutritional requirements, and the milk Ca and P concentrations found in the present study, milk can provide different age groups with 15–75% of Ca’s RNI and 15–73% of P’s RNI. The greatest contribution of milk to Ca and P’s RNIs was seen for children 1.5–3 years old because this age group has the greatest reliance on milk in their diet among in the UK population (Table 3). When compared with milk from CHP and CLP farms, milk from CSP farms provides 12.3 mg/d and 11.1 mg/d less Ca (thus reducing the contribution to RNI from 72.1% and 71.7% to 68.6%, respectively), as well as 6.2 mg/d and 7.2 mg/d less P (thus reducing contribution to RNI from 73.6% and 74.0% to 71.3%, respectively) to children 1.5–3 years old. OLP milk provides 11.0 mg/d and 9.1 mg/d less Ca to children aged 1.5–3 years old when compared with OHP and OSP milk (thus reducing contribution to RNI from 75.6% and 75.1% to 72.5%, respectively). Based on these calculations, the impact of farm grazing management on the intakes of Ca and P in relation to their RNIs of children 1.5–3 years old is minor and will be even less in adults, who have typically lower milk intakes and higher requirements in minerals. In addition, OHP milk provides 0.41 μg/d and 0.58 μg/d more Sn to children aged 1.5–3 years old than OSP and OLP milk. Sn is generally considered as a toxic element and its provisional tolerable weekly intake (PTWI) is 14 mg/kg body weight [43]. Assuming that a 2-year-old child of 12.5 kg body weight consumes 241 mL milk per day (the recorded amount for children aged 1.5–3 years old in the National Diet and Nutrition Survey), OHP, OSP and OLP milk provides 0.0040%, 0.0024% and 0.0017% of Sn’s PTWI, respectively. Considering the intakes of Sn from milk comprise negligible fractions of its PTWI, the consumption of milk and the potential impact of farm grazing management on Sn intake of children aged 1.5–3 years old is also negligible. Given that children aged 1.5–3 years old have the greatest reliance on liquid milk, as well as the lowest RNIs of minerals among all age groups, the impact of farm grazing management on mineral intakes, as a relation to their nutrient requirements, of all other age groups would be even smaller.

## 5. Conclusions

The results revealed the effects of farm grazing management, mainly with respect to pasture intake, on milk yield, basic composition and mineral concentrations in conventional and organic dairy production systems. In both production systems, milk yield showed a pattern to decrease with increasing pasture intake, which also affected milk basic composition in the organic but not in the conventional production system. Organic farms with high-pasture feeding produced milk with lower average fat and protein contents when compared with those with low- and standard-pasture feeding. In contrast, milk lactose content was higher in the high-pasture organic farms than the low-pasture organic farms. The differences in milk composition between organic farm groups were partly driven by the diet (intakes of pasture and concentrate feeds), but also breeding strategies around the use of alternative (non-Holstein) breeds which were typically seen in higher numbers in farms that fed more pasture. Farm grazing management affected the concentrations of Ca, P and Sn in milk. In conventional production systems, milk Ca and P concentrations were lower in farms with standard-pasture feeding than those with high- and low-pasture feeding. In organic production systems, farms with low-pasture feeding had a lower milk Ca concentration than those with high- and standard-pasture feeding. The differences in milk Ca and P concentrations between farm groups were likely attributed to the different proportions of non-Holstein cows in the herds, as well as diet due to the inherent association between milk protein and solids (known to be affected by cow diet) with Ca and P, respectively. Milk Sn concentration was higher in the high-pasture organic farms than the standard- and low-pasture organic farms, and the difference may have resulted from the increased grazing activity (which may be associated to increased soil ingestion compared with indoor feeding). Based on the current milk consumption rates in the UK, the differences in milk mineral concentrations between farm groups with different grazing management may only have a minor impact on consumers’ daily mineral intakes.

## Figures and Tables

**Table 1:** Breed composition and intakes of feed in conventional and organic farms with different grazing management.

Parameters Assessed	Conventional					Organic				
	Mean ^1^			SE	*p*-Value	Mean ^1^			SE	*p*-Value
	CHP *n* = 118	CSP *n* = 120	CLP *n* = 120			OHP *n* = 96	OSP *n* = 91	OLP *n* = 96		
Herd composition (% herd)										
Holstein	63.8	89.1	96.4	10.19	0.077	40.2	59.8	87.3	13.62	0.070
British Friesian	2.8	10.0	1.7	6.05	0.579	2.3	0.3	1.8	1.26	0.528
Ayrshire	0	0	0.6	0.34	0.381	12.9	12.8	0.0	9.95	0.580
New Zealand Friesian	0.5	0	0	0.24	0.371	4.2	0.0	0.0	2.42	0.387
Jersey	0.1	0	1.2	0.70	0.413	0.4	4.6	2.2	2.81	0.579
Scandinavian Red	0.4	0	0	0.20	0.371	1.0	1.1	0.8	0.74	0.944
Shorthorn	0	0	0.003	0.0019	0.384	1.0	0.1	0.6	0.36	0.271
Brown Swiss	0	0.9	0	0.55	0.381	0.3	1.0	4.9	2.90	0.490
Guernsey	0.1	0	0.1	0.10	0.563	n.a.^3^	n.a. ^3^	n.a. ^3^	n.a. ^3^	n.a. ^3^
Other breeds/crossbreds	32.4 ^a^	0 ^b^	0 ^b^	8.35	0.014	37.7	20.3	2.3	13.03	0.183
Intakes of feed (% dry matter intake unless otherwise stated)										
Dry matter intake (kg/d)	19.3 ^b^	20.8 ^a^	20.6 ^a^	0.37	0.015	17.5 ^b^	18.3 ^a,b^	19.8 ^a^	0.53	0.019
Total forage	70.2 ^a^	60.1 ^b^	54.4 ^c^	1.91	<0.001	77.7 ^a^	76.5 ^a^	68.7 ^b^	1.40	< 0.001
Total concentrate	29.8 ^c^	39.9 ^b^	45.7 ^a^	1.91	<0.001	22.3 ^b^	23.5 ^b^	31.3 ^a^	1.40	<0.001
Pasture intake	26.0 ^a^	6.2 ^b^	0.6 ^c^	1.23	<0.001	37.3 ^a^	25.1 ^b^	13.9 ^c^	1.48	<0.001
Grass/clover silage ^2^	21.8	23	22.6	2.77	0.948	32.2	35.4	35.2	2.72	0.654
Maize silage	18.6 ^b^	27.1 ^a^	27.9 ^a^	2.24	0.010	0	0	3.2	1.20	0.122
Lucerne silage	0	0	0.5	0.22	0.167	0	0.2	0	0.08	0.379
Other Mixed silage	0.1	0.5	0	0.21	0.225	1.7	3.7	3.6	1.44	0.542
Wholecrop silage	1.2	0.6	0.1	0.36	0.102	4.3 ^b^	8.9 ^a,b^	10.8 ^a^	1.71	0.038
Hay and straw	2.5	2.7	2.5	0.83	0.982	2.3	2.7	2.1	0.73	0.784
Moist by-product	2.8	3.2	6.7	1.57	0.165	0.3	0.5	0.2	0.23	0.632
Dry straight	5.0	10.4	11.2	3.66	0.436	0.5	0.3	1.5	0.42	0.136
Cereal	2.3	0.9	3.4	1.03	0.270	3.2	3.0	5.5	1.29	0.302
Blend	19.5	24.3	23.2	4.23	0.694	18.2	19.7	24.1	1.99	0.125
Oil	0.2 ^b^	1.0 ^a^	1.2 ^a^	0.24	0.014	0.03	0	0	0.017	0.391
Mineral (g/cow/day)	65.2	114.1	158.3	26.04	0.057	54.8	45.4	103.1	17.32	0.059
Vitamin (g/cow/day)	0	0.5	11.7	5.45	0.244	0	0	3.2	1.20	0.122

^1^ Predicted means from the fitted mixed linear model. ^2^ For conventional: primarily perennial ryegrass silage; for organic: mixed grass-clover silage. ^3^ not applicable: there were not Guernsey purebred or crossbred cows in the organic herds. ^a,b,c^ Significant differences between groups within production system are indicated with different letters (*p* < 0.05). CHP, conventional high-pasture feeding farms; CSP, conventional standard-pasture feeding farms; CLP, conventional low-pasture feeding farms; OHP, organic high-pasture feeding farms; OSP, organic standard-pasture feeding farms; OLP, organic low-pasture feeding farms.

**Table 2:** Means, standard errors and ANOVA *p*-values for the effect of grazing management on milk yield, basic composition, and mineral concentrations in conventional and organic farms.

Parameters Assessed	Conventional					Organic				
	Mean ^1^			SE	*p*-Value	Mean ^1^			SE	*p*-Value
	CHP *n* = 118	CSP *n* = 120	CLP *n* = 120			OHP *n* = 96	OSP *n* = 91	OLP *n* = 96		
Yield and basic composition										
Milk yield (kg/d)	26.9 ^b^	32.3 ^a^	30.2 ^a^	1.04	0.004	19.3 ^b^	21.4 ^b^	25.3 ^a^	1.04	0.002
Fat (g/kg milk)	39.9	38.4	39.1	0.44	0.085	40.3 ^a^	40.8 ^a^	38.5 ^b^	0.58	0.032
Protein (g/kg milk)	33.8	32.9	33.2	0.28	0.100	34.1 ^a^	33.6 ^a^	32.2 ^b^	0.46	0.023
Lactose (g/kg milk)	45.0	45.2	45.4	0.13	0.172	44.4 ^b^	44.8 ^a,b^	45.3 ^a^	0.19	0.019
SCC (×1000/mL milk)	162.9	149.3	143.8	10.00	0.492	167.5	121.6	132.5	11.12	0.076
Macrominerals (mg/kg milk)										
Ca	1015.7 ^a^	966.2 ^b^	1010.8 ^a^	9.77	0.001	1065.5 ^a^	1057.7 ^a^	1021.2 ^b^	9.76	0.008
K	1364.8	1352.5	1380.1	12.74	0.246	1369.2	1386.8	1378.3	14.27	0.857
Mg	95.5	95.4	97.4	1.03	0.511	96.7	95.0	92.8	1.02	0.069
Na	341.6	340.8	348.7	5.01	0.652	360.0	346.8	331.5	6.03	0.066
P	799.7 ^a^	774.8 ^b^	803.8 ^a^	8.37	0.045	817.3	810.5	794.0	8.85	0.246
Essential trace elements (μg/kg milk unless otherwise stated)										
Cu	62.1	61.9	58.4	3.52	0.815	51.7	51.2	55.8	3.72	0.198
Fe (mg/kg milk)	2.58	3.01	1.67	0.779	0.557	0.44	0.68	0.95	0.168	0.185
I	301.8	314.4	384.2	21.21	0.626	394.8	341.8	300.9	33.13	0.782
Mn	54.2	49.4	43.4	8.53	0.460	30.2	28.8	28.4	2.49	0.804
Mo	62.2	59.5	61.2	2.55	0.713	75.5	72.4	73.3	4.12	0.995
Zn (mg/kg milk)	5.07	5.07	5.10	0.210	0.989	4.78	4.25	4.50	0.238	0.255
Non-essential trace elements										
Al (mg/kg milk)	1.37	1.65	1.08	0.440	0.736	0.24	0.31	0.39	0.075	0.421
Sn (μg/kg milk)	3.26	2.60	2.10	0.394	0.128	4.05 ^a^	2.40 ^b^	1.72 ^b^	0.625	0.016

^1^ Predicted means from the fitted mixed linear model for milk yield and basic composition parameters; means of the measured values for somatic cell count (SCC) and minerals. ^2^ SCC values were log(x)-transformed and mineral parameters were log(x + 1)-transformed prior to the analysis. ^a,b^ Significant differences between groups within the production system are indicated with different letters (*p* < 0.05). CHP, conventional high-pasture feeding farms; CSP, conventional standard-pasture feeding farms; CLP, conventional low-pasture feeding farms; OHP, organic high-pasture feeding farms; OSP, organic standard-pasture feeding farms; OLP, organic low-pasture feeding farms.

**Table 3:** Example of the impact of consuming milk from different grazing management systems on the contribution to 1.5–3-year-old children’s reference nutrient intakes (RNI) or provisional tolerable weekly intake (PTWI) of minerals where a significant effect of grazing management was observed.

		RNI/PTWI % Contributed by Liquid Milk Intake		
Element	RNI/PTWI ^1^	HP	SP	LP
Conventional production system				
Ca	350 mg/d	72.1	68.6	71.7
P	270 mg/d	73.6	71.3	74.0
Organic production system				
Ca	350 mg/d	75.6	75.1	72.5
Sn	175 mg/week	0.004	0.002	0.002

^1^ The numbers refer to RNIs of Ca and P and PTWI of Sn. HP, SP and LP refer to CHP, CSP, and CLP (as defined in the manuscript CHP, conventional high-pasture feeding farms; CSP, conventional standard-pasture feeding farms; CLP, conventional low-pasture feeding farms) in conventional production systems, and to OHP, OSP, and OLP (as defined in the manuscript OHP, organic high-pasture feeding farms; OSP, organic standard-pasture feeding farms; OLP, organic low-pasture feeding farms) in organic production systems, respectively.

## Data Availability

Restrictions apply to the availability of the data used for this study and data sharing is not applicable.

## Supplementary Materials

The following are available online at https://www.mdpi.com/article/10.3390/foods10112733/s1, Table S1: Average liveweight for each breed used to estimated total dry matter intake and pasture intake (by difference), as described by Butler et al. [19]; Table S2: Changes in milk yield and basic composition over time in conventional and organic farms; Table S3: Changes in milk mineral concentrations over time in conventional farms; Table S4: Changes of milk mineral composition over time in organic farms; Figure S1: Intakes of dry matter, forage, concentrate, grazing, grass/clover silage, and maize silage in the conventional farms with different grazing management; Figure S2: Intakes of dry matter, forage, concentrate, grazing, grass/clover silage, and maize silage in the organic farms with different grazing management; Figure S3: Scatter plots of all measurements of macromineral, trace element and heavy metal concentrations in milk from the bulk tank of 30 conventional farms, collected monthly over one year; Figure S4: Scatter plots of all measurements of macromineral, trace element and heavy metal concentrations in milk from the bulk tank of 24 organic farms, collected monthly over a year.
