# Supplementary material for: Mineral Concentrations in Bovine Milk from Farms with Contrasting Grazing Management
Source: Foods. 2021 Nov 9;10(11):2733. doi: 10.3390/foods10112733 (PMC8620383; doi:10.3390/foods10112733)
Supplement: Supplementary file 1 [file foods-10-02733-s001.zip › foods-1440287-supplementary.pdf]

Supplementary material

Table S1. Average liveweight for each breed used to estimated total dry matter intake and pasture intake (by difference), as described by Butler et al. [19].

Breed	Liveweight (kg)	Source
Holstein	680	https://www.thecattlesite.com/breeds/dairy/22/holstein/
British Friesian	580	https://www.cows.ie/british-friesian-cattle/
New Zealand Friesian	468	https://www.crv4all-international.com/wp-content/uploads/2016/02/229-14-info-rassen-NZ-Genetics.pdf
Jersey	425	https://www.thecattlesite.com/breeds/dairy/23/jersey/
Scandinavian Red	548	http://www.thecattlesite.com/breeds/dairy/37/norwegian-red/
Shorthorn	600	https://www.vetstream.com/treat/bovis/breeds-pages/shorthorn
Ayrshire	544	https://www.thecattlesite.com/breeds/dairy/19/ayrshire/
Montbeliarde	685	https://www.thecattlesite.com/breeds/dairy/27/montbliarde/
Brown Swiss	590	https://www.thecattlesite.com/breeds/dairy/31/brown-swiss/
Guernsey	475	https://www.thecattlesite.com/breeds/dairy/21/guernsey/
Other breed	560	Average of all other breeds
Crossbred	560	Average of all other breeds

[a] Butler, G., Nielsen, J., Slots, T., Seal, C., Eyre, M., Sanderson, R., & Leifert, C. (2008). Fatty acid and fat-soluble antioxidant concentrations in milk from high- and low-input conventional and organic systems: seasonal variation. *Journal of Science of Food and Agriculture, 88*, 1431-1441.

Table S2. Changes in milk yield and basic composition over time in conventional and organic farms.

Parameter	Mean[1]												SE	*p*-value[2]
	Jan n = 29	Feb n = 30	Mar n = 30	Apr n = 30	May n = 30	Jun n = 30	Jul n = 30	Aug n = 30	Sep n = 30	Oct n = 30	Nov n = 30	Dec n = 29		
Conventional farms														
Milk yield (kg/d)	30.5^{ab}	29.8^{bcde}	30.8^{a}	30.4^{abc}	30.3^{abcd}	29.6^{defg}	29.9^{bcde}	29.4^{efg}	29.1^{efg}	28.7^{g}	28.9^{fg}	29.7^{cdef}	0.66	<0.001
Fat (g/kg milk)	40.6^{a}	38.9^{cd}	39.5^{bc}	38.7^{cde}	37.8^{e}	38.4^{de}	36.7^{f}	38.7^{cde}	38.6^{cde}	40.4^{ab}	40.8^{a}	40.3^{ab}	0.44	<0.001
Protein (g/kg milk)	33.6^{c}	33.3^{cd}	33.1^{de}	32.8^{ef}	32.7^{fg}	32.4^{gh}	32.2^{h}	32.6^{fg}	33.2^{d}	34.2^{b}	34.9^{a}	34.3^{b}	0.19	<0.001
Lactose (g/kg milk)	45.5^{a}	45.3^{abc}	45.5^{ab}	45.2^{cd}	45.3^{cd}	45.3^{bc}	45.2^{cde}	44.9^{fg}	44.9^{g}	45.0^{fg}	45.1^{def}	45.1^{efg}	0.09	<0.001
SCC (×1000/ml milk)	161.6^{ab}	134.7^{c}	158.7^{ab}	167.5^{ab}	156.5^{ab}	178.1^{ab}	145.8^{bc}	161.2^{ab}	122.5^{d}	145.6^{bc}	145.8^{bc}	146.1^{bc}	9.18	<0.001
Organic farms														
Milk yield (kg/d)	22.0	22.1	21.3	23.3	23.5	22.3	21.1	21.0	21.5	21.5	22.0	22.3	0.87	0.149
Fat (g/kg milk)	40.9^{abc}	39.8^{c}	40.0^{bc}	38.4^{d}	37.6^{d}	38.3^{d}	37.2^{d}	40.2^{bc}	40.9^{abc}	41.6^{a}	42.2^{a}	41.2^{ab}	0.57	<0.001
Protein (g/kg milk)	32.9^{cde}	32.8^{de}	32.5^{e}	33.3^{bcde}	33.3^{bcde}	33.1^{bcde}	32.6^{e}	33.8^{ab}	33.5^{abcd}	33.8^{abc}	34.3^{a}	33.7^{abcd}	0.41	0.002
Lactose (g/kg milk)	45.1^{abcd}	45.1^{abc}	45.5^{a}	45.3^{ab}	45.2^{abc}	44.8^{cd}	44.3^{ef}	44.1^{f}	44.3^{ef}	44.7^{de}	44.9^{bcd}	44.9^{bcd}	0.18	<0.001
SCC (×1000/ml milk)	144.7^{abc}	123.0^{c}	158.1^{ab}	134.1^{abc}	130.1^{bc}	155.3^{ab}	154.6^{ab}	164.3^{a}	108.5^{d}	141.7^{ab}	138.8^{abc}	133.6^{abc}	12.67	<0.001

[1]The means of milk yield, fat, protein, and lactose contents are the predicted means obtained from the fitted mixed linear model. The means of somatic cell count (SCC) and is the arithmetic means of the measured values.

[2]The *p*-value of SCC was obtained from the fitted mixed linear model based on log(x)-transformed values.

Different letters indicate significant difference between months ($p < 0.05$).

Table S3. Changes in milk mineral concentrations over time in conventional farms.

Mineral	Mean[1]												SE	*p*-value[2]
	Jan n = 29	Feb n = 30	Mar n = 30	Apr n = 30	May n = 30	Jun n = 30	Jul n = 30	Aug n = 30	Sep n = 30	Oct n = 30	Nov n = 30	Dec n = 29		
Macrominerals (mg/kg milk)														
Ca	952.7^{d}	966.3^{cd}	1045.9^{ab}	949.8^{de}	969.4^{cd}	912.9^{e}	1041.0^{ab}	973.0^{cd}	1061.5^{ab}	1062.6^{a}	1003.7^{bc}	1032.4^{ab}	15.87	<0.001
K	1242.4^{e}	1341.6^{d}	1412.6^{abc}	1334.9^{d}	1365.7^{bcd}	1259.9^{e}	1444.0^{a}	1353.8^{cd}	1437.4^{ab}	1438.4^{a}	1336.7^{d}	1420.9^{ab}	19.64	<0.001
Mg	89.7^{fg}	91.2^{def}	102.1^{ab}	91.3^{ef}	94.6^{cde}	86.2^{g}	98.9^{bc}	94.2^{de}	103.0^{ab}	104.8^{a}	101.4^{ab}	95.6^{cd}	1.62	<0.001
Na	279.6^{g}	313.7^{f}	384.7^{b}	355.3^{c}	331.2^{de}	312.1^{f}	411.1^{a}	326.3^{ef}	352.3^{cd}	363.7^{c}	346.4^{cd}	348.2^{cd}	7.16	<0.001
P	684.2^{fg}	777.1^{cd}	842.4^{ab}	794.6^{cd}	805.9^{bc}	727.6^{e}	832.1^{ab}	765.1^{de}	818.7^{bc}	813.3^{bc}	792.2^{cd}	859.4^{a}	12.86	<0.001
Essential trace elements (µg/kg milk unless otherwise stated)														
Cu	93.4^{b}	49.2^{c}	118.7^{a}	47.5^{b}	45.9^{b}	42.8^{cd}	78.5^{b}	78.6^{b}	37.7^{d}	44.9^{c}	48.7^{c}	43.5^{cd}	4.26	<0.001
Fe (mg/kg milk)	8.13^{a}	0.57^{d}	3.65^{b}	0.42^{d}	0.22^{d}	0.29^{def}	12.18^{bc}	1.83^{c}	0.30^{d}	0.26^{d}	1.01^{d}	0.41^{def}	0.765	<0.001
I	414.0^{a}	386.9^{abc}	464.5^{ab}	324.7^{de}	264.6^{ef}	315.0^{cde}	256.2^{f}	283.0^{def}	323.8^{cde}	362.5^{abc}	329.6^{cde}	281.2^{ef}	40.46	<0.001
Mn	119.7^{a}	27.2^{c}	116.2^{a}	24.9^{c}	20.1^{c}	20.0^{c}	125.3^{ab}	52.8^{b}	19.4^{c}	20.1^{c}	23.5^{c}	19.7^{c}	9.12	<0.001
Mo	73.4^{c}	46.9^{ef}	105.1^{a}	49.4^{def}	51.1^{de}	50.2^{def}	72.7^{c}	88.1^{b}	43.6^{f}	50.2^{def}	52.8^{d}	47.4^{def}	3.20	<0.001
Zn (mg/kg milk)	6.37^{c}	4.12^{de}	8.87^{a}	4.06^{de}	3.97^{de}	4.49^{d}	6.28^{c}	7.08^{b}	3.62^{e}	4.05^{de}	4.17^{de}	3.82^{de}	0.256	<0.001
Non-essential trace elements														
Al (mg/kg milk)	4.93^{a}	0.32^{c}	4.76^{b}	0.27^{c}	0.07^{c}	0.08^{c}	4.47^{b}	0.64^{c}	0.20^{c}	0.14^{c}	0.40^{c}	0.17^{c}	0.464	<0.001
Sn (µg/kg milk)	4.64^{ab}	1.51^{d}	4.79^{a}	1.85^{cd}	3.63^{abc}	1.59^{d}	2.32^{bcd}	1.94^{cd}	1.40^{d}	2.04^{d}	1.72^{d}	4.49^{a}	0.657	0.004

[1]Arithmetic means of the measured values.
[2]The *p*-values were obtained from the fitted mixed linear model based on the log(x+1) transformed values.
Different letters indicate significant difference between months ($p < 0.05$).

Table S4. Changes of milk mineral composition over time in organic farms.

Mineral	Mean[1]												SE	p-value[2]
	Jan n = 29	Feb n = 30	Mar n = 30	Apr n = 30	May n = 30	Jun n = 30	Jul n = 30	Aug n = 30	Sep n = 30	Oct n = 30	Nov n = 30	Dec n = 29		
Macrominerals (mg/kg milk)														
Ca	975.0^{cd}	1001.4^{c}	1081.4^{b}	1010.0^{c}	1009.3^{c}	952.2^{d}	1096.5^{b}	1068.9^{b}	1083.6^{b}	1147.8^{a}	1091.5^{b}	1060.1^{b}	16.06	<0.001
K	1178.9^{g}	1305.6^{e}	1374.7^{cd}	1349.1^{de}	1383.3^{cd}	1247.4^{f}	1478.5^{ab}	1430.6^{bc}	1430.0^{bc}	1527.0^{a}	1412.1^{cd}	1418.3^{c}	20.52	<0.001
Mg	85.3^{de}	91.4^{c}	99.6^{b}	89.0^{cd}	92.2^{c}	85.4^{e}	100.1^{b}	98.0^{b}	97.9^{b}	104.9^{a}	102.1^{ab}	92.1^{c}	1.58	<0.001
Na	265.0^{e}	323.1^{de}	369.6^{b}	354.2^{bc}	332.6^{cde}	314.7^{d}	421.3^{a}	357.8^{bc}	356.8^{bc}	367.1^{b}	348.5^{bc}	343.8^{c}	9.65	<0.001
P	648.1^{f}	774.3^{d}	835.9^{abc}	837.7^{abc}	836.9^{abc}	740.0^{e}	856.5^{ab}	827.4^{bc}	808.2^{cd}	837.7^{abc}	823.4^{c}	865.0^{a}	12.73	<0.001
Essential trace elements (µg/kg milk unless otherwise stated)														
Cu	44.7^{bcd}	107.4^{a}	99.7^{a}	47.8^{bc}	35.9^{de}	36.0^{cde}	31.6^{e}	43.5^{bcd}	49.0^{b}	43.5^{bc}	50.8^{b}	48.8^{b}	4.54	<0.001
Fe (mg/kg milk)	1.83^{ab}	1.21^{a}	0.74^{bc}	0.33^{de}	0.20^{e}	0.25^{de}	1.86^{abc}	0.64^{cd}	0.32^{de}	0.22^{e}	0.34^{de}	0.25^{de}	0.197	<0.001
I	442.7^{ab}	383.7^{a}	479.4^{a}	319.1^{cd}	243.6^{ef}	253.1^{def}	237.1^{f}	275.6^{de}	328.6^{bc}	426.3^{a}	392.0^{ab}	372.9^{ab}	71.79	<0.001
Mn	38.0^{c}	71.4^{a}	46.8^{b}	22.2^{de}	17.5^{e}	20.6^{de}	29.5^{d}	26.1^{d}	21.6^{de}	17.6^{e}	20.8^{de}	19.2^{de}	3.14	<0.001
Mo	57.8^{e}	144.6^{a}	107.0^{b}	66.8^{cdef}	58.9^{e}	59.3^{e}	47.2^{f}	63.9^{de}	66.1^{cde}	66.7^{cde}	78.4^{c}	73.5^{cd}	5.70	<0.001
Zn (mg/kg milk)	3.72^{de}	8.93^{a}	7.25^{b}	3.90^{cd}	3.38^{de}	4.27^{c}	3.19^{e}	4.09^{cd}	4.36^{c}	3.53^{de}	3.95^{cd}	3.89^{cd}	0.264	<0.001
Non-essential trace elements														
Al (mg/kg milk)	0.83^{ab}	0.80^{a}	0.44^{bc}	0.17^{de}	0.06^{e}	0.11^{de}	0.53^{cd}	0.24^{cde}	0.17^{de}	0.11^{de}	0.18^{cde}	0.11^{de}	0.103	<0.001
Sn (µg/kg milk)	2.19^{bcd}	3.63^{a}	3.40^{ab}	2.08^{bcde}	2.49^{abc}	1.52^{de}	5.26^{e}	1.58^{cde}	2.81^{de}	1.20^{de}	1.77^{bcde}	4.83^{ab}	0.951	<0.001

[1]Arithmetic means of the measured values.

[2]The p-values were obtained from the fitted mixed linear model based on the log(x+1) transformed values.

Different letters indicate significant difference between months ($p < 0.05$).

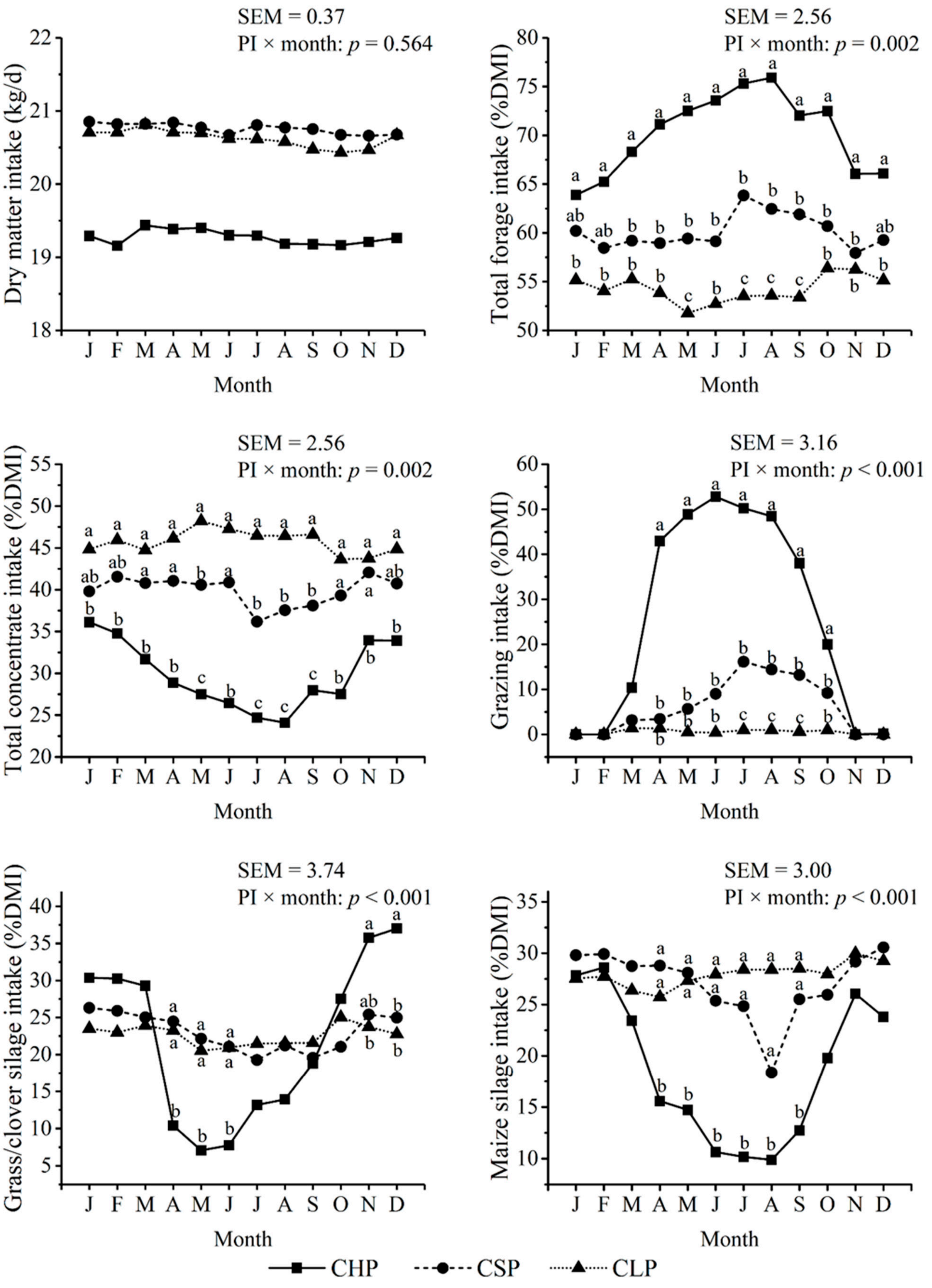

Figure S1. Intakes of dry matter, forage, concentrate, grazing, grass/clover silage, and maize silage in the conventional farms with different grazing managements. Means are the predicted means from the fitted mixed linear model. Different letters indicate significant difference between groups within months ($p < 0.05$). CHP, conventional high-pasture feeding farms; CSP, conventional standard-pasture feeding farms; CLP, conventional low-pasture feeding farms.

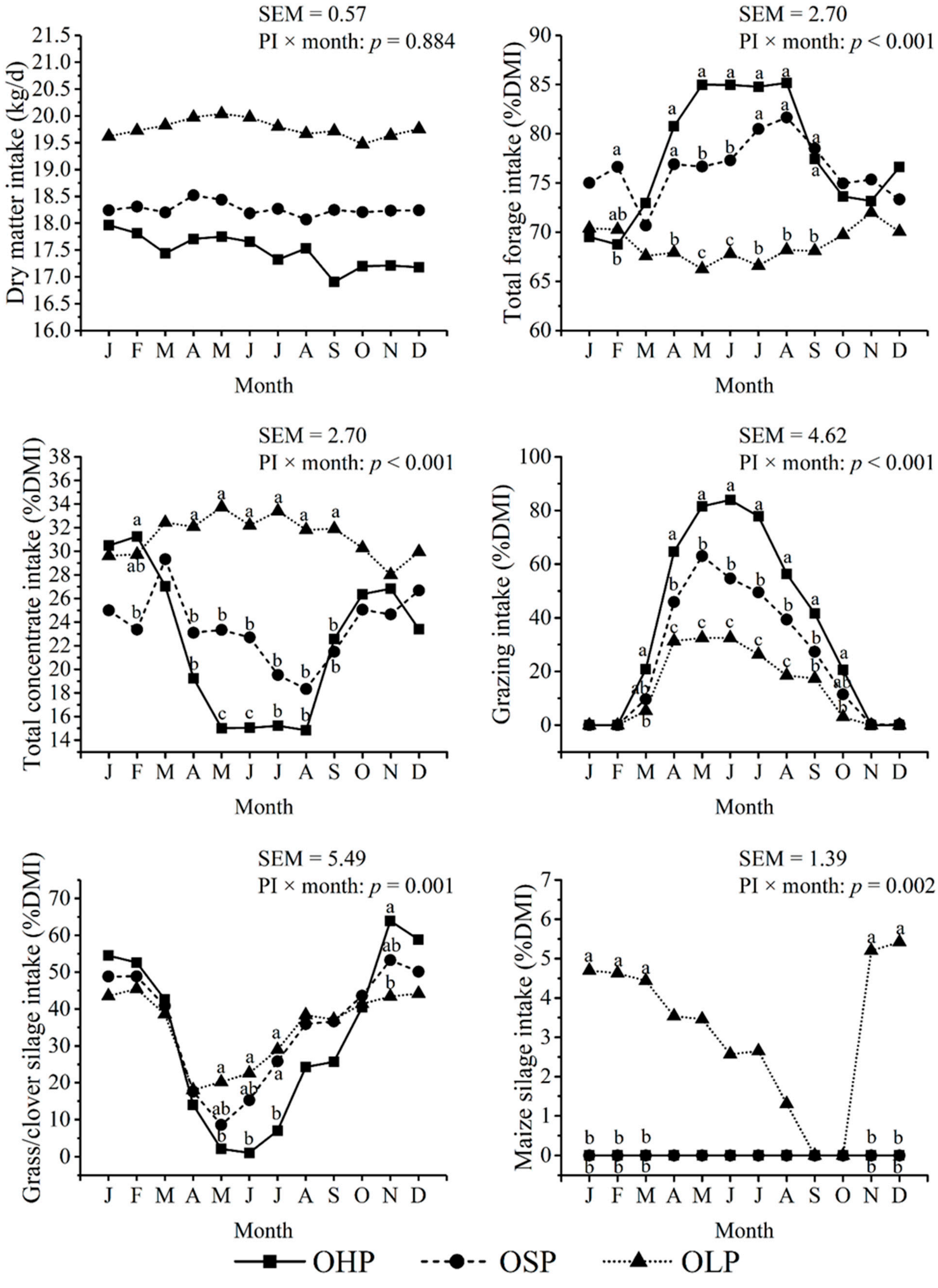

Figure S2. Intakes of dry matter, forage, concentrate, grazing, grass/clover silage, and maize silage in the organic farms with different grazing managements. Means are the predicted means from the fitted mixed linear model. Different letters indicate significant difference between groups within months ($p < 0.05$). OHP, organic high-pasture feeding farms; OSP, organic standard-pasture feeding farms; OLP, organic low-pasture feeding farms.

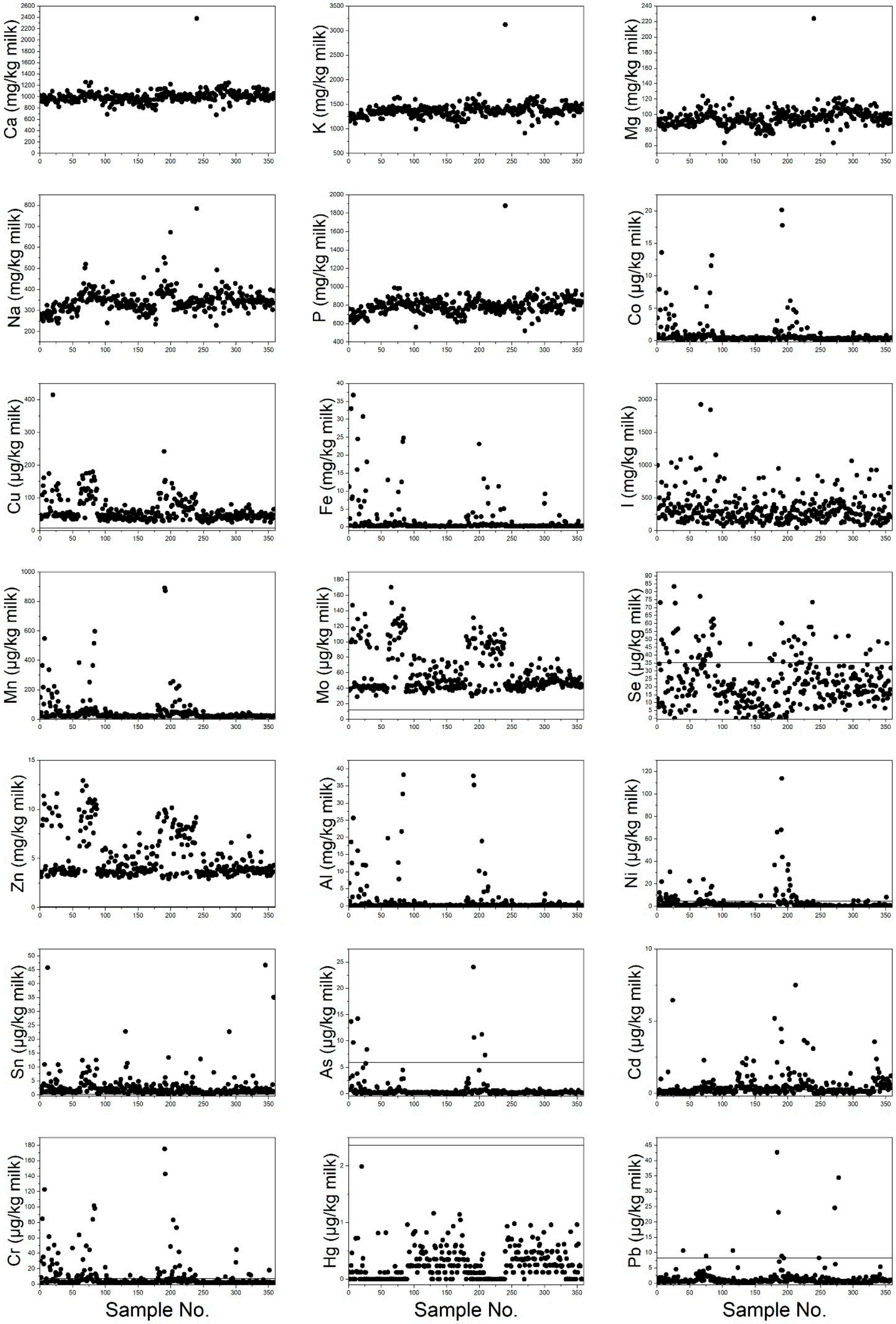
Ca (mg/kg milk)
K (mg/kg milk)
Mg (mg/kg milk)
Na (mg/kg milk)
P (mg/kg milk)
Co (μg/kg milk)
Cu (μg/kg milk)
Fe (mg/kg milk)
I (mg/kg milk)
Mn (μg/kg milk)
Mo (μg/kg milk)
Se (μg/kg milk)
Zn (mg/kg milk)
Al (mg/kg milk)
Ni (μg/kg milk)
Sn (μg/kg milk)
As (μg/kg milk)
Cd (μg/kg milk)
Cr (μg/kg milk)
Hg (μg/kg milk)
Pb (μg/kg milk)
Sample No.
Sample No.
Sample No.

Figure S3. Scatter plots of all measurements of macromineral and traces element concentrations from 30 conventional herds collected monthly over one year. The horizontal lines represent limits of quantification. The limits of quantification of trace elements were: Co, 0.59 µg/kg milk; Cu, 8.26 µg/kg milk; Fe, 88.45 µg/kg milk; Mn, 5.90 µg/kg milk; Mo, 11.79 µg/kg milk; Se, 35.38 µg/kg milk; Zn, 82.56 µg/kg milk; Al, 23.59 µg/kg milk; Ni, 4.72 µg/kg milk; Sn, 0.24 µg/kg milk; As, 5.90 µg/kg milk; Cd, 0.24 µg/kg milk; Cr, 7.08 µg/kg milk; Hg, 2.36 µg/kg milk; Pb, 8.26 µg/kg milk. The proportions of individual measurements which were below LOQs were: Co, 74%; Cu, 0%; Fe, 0%; Mn, 0%; Mo, 0%; Se, 83%; Zn, 0%; Al, 4%; Ni, 84%; Sn, 2%; As, 97%; Cd, 56%; Cr, 77%; Hg, 99%; Pb 97%.

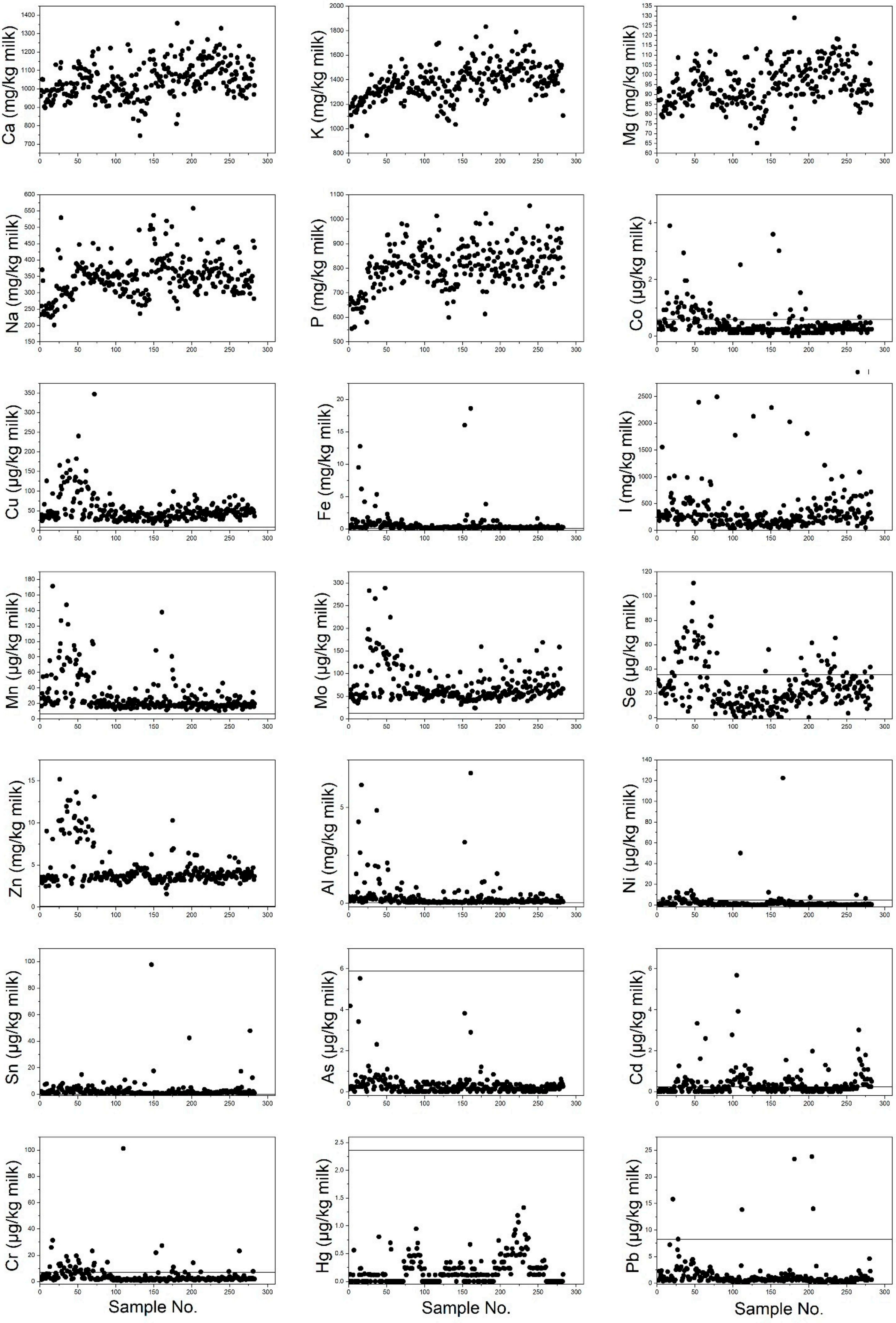
Ca (mg/kg milk)
K (mg/kg milk)
Mg (mg/kg milk)
Na (mg/kg milk)
P (mg/kg milk)
Co (µg/kg milk)
Cu (µg/kg milk)
Fe (mg/kg milk)
I (mg/kg milk)
Mn (µg/kg milk)
Mo (µg/kg milk)
Se (µg/kg milk)
Zn (mg/kg milk)
Al (mg/kg milk)
Ni (µg/kg milk)
Sn (µg/kg milk)
As (µg/kg milk)
Cd (µg/kg milk)
Cr (µg/kg milk)
Hg (µg/kg milk)
Pb (µg/kg milk)
Sample No.
Sample No.
Sample No.

Figure S4. Scatter plots of all measurements of macromineral and traces element concentrations in milk from 24 organic herds collected monthly over a year. The horizontal lines represent limits of quantification. The limits of quantification of trace elements were: Co, 0.59 µg/kg milk; Cu, 8.26 µg/kg milk; Fe, 88.45 µg/kg milk; Mn, 5.90 µg/kg milk; Mo, 11.79 µg/kg milk; Se, 35.38 µg/kg milk; Zn, 82.56 µg/kg milk; Al, 23.59 µg/kg milk; Ni, 4.72 µg/kg milk; Sn, 0.24 µg/kg milk; As, 5.90 µg/kg milk; Cd, 0.24 µg/kg milk; Cr, 7.08 µg/kg milk; Hg, 2.36 µg/kg milk; Pb, 8.26 µg/kg milk. The proportions of individual measurements which were below LOQs were: Co, 81%; Cu, 0%; Fe, 0%; Mn, 0%; Mo, 0%; Se, 80%; Zn, 0%; Al, 1%; Ni, 89%; Sn, 5%; As, 98%; Cd, 63%; Cr, 82%; Hg, 100%; Pb 96%.
